# Small molecule inhibitor of Igf2bp1 represses Kras and a pro-oncogenic phenotype in cancer cells

**DOI:** 10.1080/15476286.2021.2010983

**Published:** 2021-12-11

**Authors:** Nadav Wallis, Froma Oberman, Khriesto Shurrush, Nicolas Germain, Gila Greenwald, Tehila Gershon, Talia Pearl, Giancarlo Abis, Vikash Singh, Amandeep Singh, Arun K. Sharma, Haim M. Barr, Andres Ramos, Vladimir S. Spiegelman, Joel K. Yisraeli

**Affiliations:** a Department of Developmental Biology and Cancer Research, IMRIC, Faculty of Medicine, Hebrew University of Jerusalem, Jerusalem, Israel; b The Wohl Drug Discovery Institute of the Nancy and Stephen Grand Israel National Center for Personalized Medicine, Weizmann Institute of Science, Rehovot, Israel; c Division of Biosciences, Institute of Structural and Molecular Biology, University College London, London, UK; d Department of Pediatrics, Division of Pediatric Hematology/Oncology, Pennsylvania State University, College of Medicine, Hershey, PA, USA; e Department of Pharmacology, Penn State Cancer Institute, Penn State College of Medicine, Hershey, PA, USA

**Keywords:** Fluorescence polarization, RNA binding proteins, Kras inhibitor, Igf2bps

## Abstract

Igf2bp1 is an oncofetal RNA binding protein whose expression in numerous types of cancers is associated with upregulation of key pro-oncogenic RNAs, poor prognosis, and reduced survival. Importantly, Igf2bp1 synergizes with mutations in Kras to enhance signalling and oncogenic activity, suggesting that molecules inhibiting Igf2bp1 could have therapeutic potential. Here, we isolate a small molecule that interacts with a hydrophobic surface at the boundary of Igf2bp1 KH3 and KH4 domains, and inhibits binding to Kras RNA. In cells, the compound reduces the level of Kras and other Igf2bp1 mRNA targets, lowers Kras protein, and inhibits downstream signalling, wound healing, and growth in soft agar, all in the absence of any toxicity. This work presents an avenue for improving the prognosis of Igf2bp1-expressing tumours in lung, and potentially other, cancer(s).

## Introduction

The IGF2BP (*insulin-like growth factor 2 mRNA binding protein*) family of RNA-binding proteins consists of three highly related (~80% similarity) paralogues that contain six RNA binding domains and regulate stability, localization, translation, and/or alternative splicing of their mRNA targets [1]. Igf2bp1 and Igf2bp3 are oncofetal proteins, expressed in a variety of embryonic tissues, downregulated or silenced after birth, but expressed at high levels in patients with many different types of solid and blood cancers [2–13]. Igf2bp2 is expressed not only during embryonic development but also into adulthood. This paralogue is associated as well with many different types of cancers, often expressed in conjunction with one or more of the other paralogues [14]. We have recently demonstrated that Igf2bp1 can cooperate in the induction of lung adenocarcinoma (LUAD) and melanoma metastasis, and down-regulation of Igf2bp1 severely inhibits metastasis in diverse immunocompetent mouse models of LUAD and melanoma progression [3,11]. Additionally, we have shown that inhibition of Igf2bp1 sensitizes melanoma to chemotherapy and targeted therapies [8,15]. Igf2bp1 appears to mediate many of its pro-oncogenic effects by binding a wide variety of cancer promoting RNAs, protecting them from degradation, enhancing their translation, and/or regulating their intracellular localization [16].

Ras oncogenes are the most common site for aberrations in human cancers, and the Kras gene is the most commonly mutated among the three Ras genes [17]. Kras has proven to be an extremely difficult protein to target, and there are currently no Kras-targeting agents approved for clinical use. Recently, much excitement has focused on several covalent allosteric inhibitors that bind to a shallow pocket on a Kras mutant commonly found in cancer, Kras^G12C^, and these inhibitors are currently in early-phase studies. Nevertheless, many challenges remain, including how to target other Kras mutants and prevent the development of resistance to these drugs [17]. Strategies combining different approaches are likely to be crucial for effective treatment.

Igf2bp1 was previously shown to bind Kras RNA [10,18] and synergize with a Kras mutation, inducing the formation of large tumours [11]. Consistent with these results, lung carcinoma patients expressing elevated Igf2bp1 levels along with mutant Kras have a strikingly reduced expected survival as compared to those patients with low levels of Igf2bp1 (15.45 months as opposed to 88.17 months, respectively, with a hazard ratio of 4.753). Furthermore, the use of a dominant negative construct to inhibit Igf2bp1 RNA binding strongly reduced metastasis formation in a murine xenograft model. Thus, a drug that could inhibit Igf2bp1 binding to Kras, and possibly other, pro-oncogenic RNAs, could be a novel and powerful tool in a clinical setting for treating many cancers.

Small-molecule inhibitors are receiving increased attention for use in directed therapies. Several RNA binding proteins (RBPs) have been targeted and are at various stages of clinical testing [19]. To identify an efficient Igf2bp1 inhibitor, we performed a fluorescent polarization (FP)-based high throughput screen (HTS) of over 27,000 small molecules, using a Kras RNA fragment as a probe, and the most promising candidates were further validated by *in vitro* and cell-based assays. We report here the identification of a compound, termed ‘7773’, that directly binds Igf2bp1 and inhibits its binding to Kras RNA. By directly targeting Igf2bp1 in cells, 7773 reduces the level of Kras and other mRNAs, lowers Kras protein and downstream signalling, and inhibits Igf2bp1 pro-oncogenic activity.

## Material and methods

### RNA probe synthesis

Kras fragments for the 3ʹUTR RNA probes were synthesized by PCR amplifications of a pGEM-T_Easy plasmid containing part of the Kras cDNA, using the following primer sets:

**Table:** 

	F primer	R primer	Length (bp)	Position/region (Ref sequence-NM_004985)
Kras1	GCTT**TAATACGACTCACTATAGGG**ATGACTGAATATAAACTTGT	ATTGCACTGTACTCCTCATGA	200	191/CDS
Kras2	GCTT**TAATACGACTCACTATAGGG**TGAGGAGTACAGTGCAATGA	TTGTGTCTACTGTTCTAGAAG	199	372/CDS
Kras3	GCTT**TAATACGACTCACTATAGGG**GAACAGTAGACACAAAACAG	TTACATAATTACACACTTTGT	200	557/CDS
Kras4	GCTT**TAATACGACTCACTATAGGG**ATACAATTTGTACTTTTTTC	AAACTCTGGGAATACTGGCAC	200	758/3ʹUTR
Kras5	GCTT**TAATACGACTCACTATAGGG**AAATGCTTATTTTAAAATGA	TTAATTTGTTTCACACCAAC	200	892/3ʹUTR
Kras6	GCTT**TAATACGACTCACTATAGGG**GTGCATGCAGTTGATTACTT	GCTTTAATACGACTCACTATAGGGGTGCATGCAGTTGATTACTT	200	1027/3ʹUTR

The amplified DNA fragments contain overlapping sequences of approximately 200bp from the Kras-coding region and 5ʹ end of the 3ʹUTR with a T7 promoter (in bold). These fragments were then used as templates in *in vitro* transcription reactions, using the MEGA Script T7 High Yield Transcription kit (Invitrogen). For FP experiments, the 5ʹEndTag Nucleic Acid Labelling System (Vector Laboratories) was used to label the RNA with fluorescein at its 5′end.BclRNAoligo/5ʹ6FAM/CCCGUUGCUUUUCCUCU GGGAAGGAUGGCGCACGCUGGG, was synthesized and HPLC purified by IDT.

### Fluorescence polarization (FP)

For the small-molecule screen, compounds were obtained from the collection maintained at the Grand Israel National Center for Personalized Medicine, consisting of molecules purchased from commercial vendors. Labelled RNA (10 nM) was incubated with recombinant Igf2bp1 protein (400 nM) in FAM-RNA buffer (10 mM Tris pH 8.0, 75 mM KCl, 0.5 mM EDTA, 1 mM DTT, 0.0188 U/μL RNAsin, 0.0005 μg/mL heparin, 0.0001 μg/μL tRNA, 0.01% Triton X-100, 3 mM MgCl_2_, 0.05% IGEPAL) at room temperature for 15 min. 7 μL of this reaction mix was aliquoted with GNF WDII (USA) into wells of 1536 well plates, with a final concentration of 20 μM compound or DMSO. The plates were spun down and read in a BMG Pherastar FS (Germany) plate reader, in FP mode with a 485/520/520 polarization filter, calibrated to 35 mP for free fluorescein with similar total intensity as experimental samples. The G9a FP assay was based on using an S-adenosylhomocysteine (SAH)-FITC probe and antibody to SAH to produce a high FP signal when the antibody binds to SAH-FITC. Unlabelled SAH produced by incubation of G9a enzyme with S-adenosylmethionine (SAM) and peptide substrate competes for the antibody and results in a low FP signal. One hundred per cent activity means that G9a is fully active and therefore a low FP signal is observed (SAH is produced)

### FP data analysis and hit scoring

To determine the quality of our screening assay, the Z’ factor for each plate was calculated with a Z’ factor greater than 0.5 indicating a robust assay suitable for high throughput screening. Compounds that altered the overall fluorescence intensity compared to controls were considered auto florescence and excluded from further analysis. Inhibition was evaluated by calculating percent inhibition relative to the control wells, where % inhibition = (1−((mPComp − mPMin)/(mPMax − mPMin))) ×100, where the assay minimum (mPMin) is fluorescence Kras RNA alone and the assay maximum (mAMax) is fluorescence Kras with Igf2bp1 protein. Any small molecule that causes a change of more than three standard deviations from the mean is determined appropriate for further investigation.

### Microscale thermophoresis (MST)

Recombinant full-length Igf2bp1 protein was labelled with RED-tris-NTA dye using a Monolith NT His-Tag Labelling Kit (NanoTemper) while RRM12, KH12 and KH34 di-domain peptides were labelled using the Protein Labelling Kit RED NHS 2^nd^ generation (NanoTemper). Labelled proteins were spun at 15,000 g for 5 min to eliminate aggregates. All samples were 2X labelled protein mix of Igf2bp1 (200 nM) in PBST and were combined (1:1) with serial dilutions of compound or Kras6 RNA in binding buffer (10mMTris pH = 7.5, 100 mM NaCl, 0.1 mM EDTA, 0.06% IPEGAL, 0.02 μg/μL tRNA, 2 mM DTT, 0.1 μg/μL heparin) in final volume of 20 μL, room temperature for 15 min. Samples were then loaded into standard capillaries (NanoTemper) and read in the Monolith NT.115 scanner (NanoTemper) using the red filter with reading conditions of 95% LED power and 40% MST power, 5 sec fluorescence before, 30 sec MST on, 5 sec fluorescence after and 25 sec delay between samples. Results were analysed using the MO Control software (NanoTemper) for curve fitting to K_D_.

### Electrophoretic mobility shift assay (EMSA)

Recombinant Igf2bp1 protein was serially diluted (1:1) starting at an initial concentration of 10,000 nM in 0.75 μg heparin, in a total volume of 13 μL. Fluorescently labelled Kras 6 RNA (750 nM) diluted in reaction buffer (10 mM Tris pH 7.5, 100 mM NaCl, 0.1 mM EDTA, 1 mg/mL tRNA, 0.25% IGEPAL (Sigma), 0.5 mM DTT) was heated to 65°C for 5 minutes, kept on ice for 1 minute and RNasin (200 U/mL) (ThermoScientific, RiboLock RNase inhibitor) was then added. 2 μL RNA was added to each well protein sample, and after a 30 min incubation at room temperature in the dark, loading dye (3 μL) (30% glycerol, 0.01% bromocresol green) was added to each mixture. For compound inhibition experiments, compounds or DMSO were incubated for 4 hours at 37 °C with the protein prior to adding the RNA. Samples were electrophoresed on native TBE polyacrylamide gels (6% acrylamide/bis-acrylamide 37.5:1, 0.5X TBE) that had been pre-run for 30 min. Gels were run at 120 V in 0.5X TBE, at 4°C in the dark. They were then carefully washed and scanned using a Typhoon FLA9500 imager using Alexa 488 laser and LPB1/2 filter. Igf2bp1-RNA complex formation was quantified using the ImageJ software. Prism6 software was used for curve fitting and K_D_ calculations.

### Tissue culture

H1299 cells were maintained in RPMI medium, and ES2, RKO, LKR-M-Fl, LKR-M-GFP, and HEK293 cells were maintained in DMEM (Biological Industries – Israel). Both media contained 10% FCS (Biological Industries) and 10 μg/mL ciprofloxacin (Bayer).

### Cell migration assay

Cells were seeded in 96 IncuCyte® ImageLock plates (20X10^3 cells per well) for 24 hours, to a near confluency of 95%, before the addition of increasing concentrations of compound. After a further 24 hours, wells were scratched using the IncuCyte® 96-well WoundMaker Tool, and the cells cultured for an additional 48 hours using the IncuCyte® S3 Live-Cell Analysis System (Essen BioScience). The plate was imaged at increments of 120 minutes for a period of 48 hours, and then analysed for relative wound healing.

### Cell proliferation assay

ES2, H1299 and HEK293 cells were seeded in a 96 well plate (5X10^3 cells per well) for 24 hours before adding the compound at increasing concentrations. After an additional 24 hours, the plate was incubated in an IncuCyte® S3 Live-Cell Analysis System (Essen BioScience). The wells were filmed for 72 hours, and then analysed for proliferation rate Using IncuCyte® S3 Live-Cell Analysis System (Essen BioScience)

### Split-luciferase assay

Vectors expressing different fragments of split luciferase reporters with flexible linker were procured from Addgene (catalogue # 58786, 58787, 58788 and 58789) [20]. The vectors were digested with EcoRI and XhoI. EcoRI and XhoI restriction enzyme sites were added to 5′ and 3′ ends of the coding region of IGF2BP1, respectively, using PCR. We constructed four split luciferase reporter vectors by cloning IGF2BP1 in different orientations with the luciferase fragments . Primers used for the amplification of IGF2BP1 were:

IGF2BP1 Forward Primer- CCGGAATTCGCCACCATGA ACAAGCTTTACATCGGCAAC

IGF2BP1 Reverse Primer without STOP Codon- CCGCTCGAGCTTCCTCCGTGCCTGGGCCTGGTT

IGF2BP1 Reverse Primer with STOP Codon- CCGCTCGAGTCACTTCCTCCGTGCCTGGGCCTG

The RKO cells were seeded in DMEM with 10% calf serum 24 hrs prior to the transfection. Transfection was done using Lipofectamine 2000 with reporter plasmids and Renilla-luciferase expressing vector (as internal control). After 24 hrs, cells were lysed using passive lysis buffer (Promega catalogue # E1941). The lysates were cleared by centrifugation and its 20 μL volume was mixed with 100 μL of luciferase substrate solution for luminescence recording which was followed by addition of 100 μL of Stop-glo solution (Promega catalogue # E1910) with an additional luminescence reading. Both readings were detected by using GloMax 20/20 Luminometer (Promega), and the final value for each sample was the ratio of their two respective readings. To test the effects of 7773, transfected cells with reporter plasmids were washed with PBS after 24 hrs of transfection and incubated with medium containing 7773.

### Western blot analysis

Western blots were performed as described [11]. For fluorescent westerns, cell lysates were prepared with phosphatase inhibitors (β-Glycerophosphate and Sodium orthovanadate) and a protease inhibitor (cOmplete™, Mini Protease Inhibitor Cocktail, Roche 04693124001), membranes were read using a Li-Core Odyssey laser scanner, and results analysed using Image Studio Lite software.

The following primary antibodies were used: mouse anti-total ERK (p44/42 MAPK (Erk1/2) L34 F12 Cell Signalling 4696s), mouse anti-Kras (CPTC-KRAS4B-2, DSHB) rabbit anti-dpERK, (p44/42 MAPK, Cell Signalling 4370), rabbit anti-alpha/beta tubulin (Cell Signalling 2148), goat anti-rabbit IgG-HRP (Jackson), goat anti-mouse IgG-HRP (Jackson). Secondary antibodies for fluorescent westerns: donkey anti-mouse 800 (Rockland 610–732-124), goat anti-rabbit 680 (Molecular Probes A21076).

### qPCR

Primers were calibrated for several Igf2bp1 RNA targets:

**Table:** 

	F primer	R primer
Kras	ACCCACCTTGGCCTCATAAAC	ACTGGCATCTGGTAGGCACTC
c-Myc	GCTGCTTAGACGCTGGATTT	GTCGAGGTCATAGTTCCTGTTG
CD44	CTCTGCGGGCTGCTTAGT	TTTATTCGAGGTTGAAAACAGTGA
SRF	CCTTTCCCATCACCAACTACCT	GCCGCTGCCTGTACTCTTC
HPRT	GGATTTGGAAAGGGTGTTTATTC	TCCCATCTCCTTCATCACATC

Cells were grown for 12 hours in 12 well plates prior to incubation with the compound at different concentrations for an additional 12 or 24 hours. Total RNA was then extracted using EZ-RNA total RNA isolation kit (Biological Industries) and cDNA prepared from the RNA using the First Strand cDNA Synthesis kit (Quanta bio). Real time PCR was performed with Fast SYBR Green Master Mix (Thermo Fisher Scientific), and cDNA expression analysed with the Bio-Rad CFX Manager 3.1.

### Growth in soft agar

Cells were grown for 12 hours in 6 well plates prior to incubation with the compound or DMSO for an additional 48 hours, 2000 cells were suspended in 0.3% agar-complete medium and then seeded in triplicates onto a 0.6% agar-complete medium base in a 6 well plate. After one week, the colonies were fed with 0.3% agar-complete medium, and after 2 additional weeks, imaged and quantified.

### Cloning

The open reading frame of Igf2bps were cloned into the pET-21(d) plasmid (Novagen) between the Nco1 and Xho1 sites, as previously described [12]. Human Igf2bp1 di-domains RRM12 (M1-P188) and KH34 (P387-A574) cDNA sequences were PCR-amplified from a pCMV6-Entry vector containing the full-length sequence (Origene – NCBI reference sequence NM_006546.4), using the KOD Hot Start Master Mix (Novagen) and primers incorporating NcoI/XhoI restriction sites. After 1–3 h digestion at 37°C with NcoI/XhoI (NEB) the cDNA sequences were ligated into the bacterial expression vector pETM11 (EMBL) overnight at 16°C using T4 DNA ligase (NEB) (insert-to-plasmid ratios of 4:1 and 3:1 for RRM12 and KH34 respectively). Plasmids were amplified in DH5α C2987 *E. coli* (NEB) and successful cloning was confirmed by sequencing (Source Bioscience). Igf2bp1 di-domain KH12 (V194-N369) cDNA sequence was previously cloned in pETM30 (EMBL) [21].

### Protein expression and purification

To induce expression of recombinant Igf2bps-His tag proteins, a single transformed BL21(DE3) colony was grown in LB medium supplemented with ampicillin (100 g/ml) at 37°C for 7 hours, with agitation. This starter was then transferred to 500 ml fresh LB and incubated with agitation for 1 hour. Protein expression was induced by the addition of IPTG to a final concentration of 0.5 mM for an additional 3 hours. Cells were harvested by centrifugation at 4700 rpm for 20 min at 4°C. The pellet was suspended in HNTA buffer (1 M NaCl, 50 mM NaPi buffer pH = 7.8 (0.9 M Na2HPO4, 0.1 M NaH2PO4), 1% TritonX-100) and Protease Inhibitor Complex (1:100) (APExBIO). Samples were lysed by sonication and spun at 13,000 RPM in cold for 20 min. The collected supernatant was incubated for 2 hours on a high speed rotator at 4°C with 10 mM imidazole and 0.5 mL Nickel beads (Adar Biotech) which had been prewashed 3X in NTA buffer (0.3 M NaCl, 50 mM NaPi buffer pH = 7.8, 1% TritionX-100). The nickel beads were then loaded on a Poly-Prep Chromatography Column (BioRad) and the recombinant protein was eluted through sequential washes of imidazole (1 ml), at the following concentrations: 20 mM, 40 mM, 50 mM, and 250 mM. Samples from all steps of the protein production process were collected and analysed by SDS-polyacrylamide gel electrophoresis followed by Coomassie blue staining. The fractions which contained the protein were dialysed at 4°C overnight using SnakeSkin Dialysis Tubing, 1000 MWCO (Thermo Scientific) in TGKED buffer (50 mM Tris pH = 7.5, 50 mM KCL, 0.1 mM EDTA, 0.5 mM DTT). Glycerol (20%v/v) was added to the protein following the dialysis.

The three Igf2bp1 di-domains RRM12, KH12 and KH34 were expressed in BL21(DE3) *E. coli* cells (Invitrogen). ^15^N-labelled N-6xHis-RRM12 and -KH34 and N-6xHis/GST-KH12 fusion proteins were obtained by growing the cells in M9 minimal media supplemented with ^15^NH_4_Cl as the sole nitrogen source. Cells were cultured at 37°C and expression was induced overnight at 18°C by adding 0.5 mM Isopropyl β-d-1-thiogalactopyranoside. After cell lysis and sonication in 10 mM TRIS-Base pH 8.0, 10 mM imidazole, 1 M NaCl, 5% glycerol, 2 mM 2-Mercaptoethanol, a tablet of cOmplete™ protease inhibitor cocktail (Roche) per 50 mL of buffer, 0.01% Triton-X, 200 μg/mL lysozyme (Sigma), KH12 and KH34 were initially purified by immobilized metal affinity chromatography (IMAC) using a HisTrap™ FF Nickel Sepharose Column (GE Healthcare). RRM12 expressing cells were lysed and sonicated in the same lysis buffer as above added with 8 M urea. Initial purification was performed with a nitrilotriacetic acid agarose matrix (ThermoFisher Scientific) using the same buffers used in IMAC added with 8 M urea. Refolding was obtained by step-wise dialysis at 4°C. After this first step of purification, the tags were removed from the three proteins by overnight cleavage with 5 µM TEV protease at 4°C in 50 mM TRIS-Base pH 7.5, 150 mM NaCl, 2 mM 2-Mercaptoethanol. The proteins were then loaded onto a cation-exchange Hi-Load SP-Sepharose 26/10 column (GE Healthcare) and eluted by applying a 0–100% gradient of 10 mM TRIS-Base, 1 M NaCl, 2 mM 2-Mercaptoethanol (pH 6.25 for RRM12 and pH 7.3 for KH12 and KH34). The final step of purification was performed using a Hi-Load 16/600 Superdex 75 pg (GE Healthcare), equilibrated with 10 mM Na_2_HPO_4_ pH 7.4, 50 mM NaCl, 1 mM tris(2-carboxyethyl)phosphine) (TCEP). The purity of the purified protein peak was assessed using SDS-PAGE [22], whilst concentration adjusted according to sample absorbance at 280 nm and theoretical extinction coefficient calculated by ProtParam ExPASy [23]. The >95% pure protein samples were stored at −80°C for use in MSP and NMR assays.

A La protein plasmid was graciously provided by Tilman Helse and purification was performed as described [24].

### Nuclear magnetic resonance (NMR) spectroscopy

NMR experiments were recorded at 25°C on a Bruker Avance spectrometer operating at 800 MHz ^1^H frequency. ^1^H-^15^N-Heteronuclear Single Quantum Coherence Nuclear Magnetic Resonance (^15^N-HSQC) experiments [25] were performed by adding the 7773 compound (in DMSO) into 50 µM samples of ^15^N-RRM12, ^15^N-KH12 and ^15^N-KH34, obtaining protein-to-7773 molar ratios of 1:1, 2, 4, 8, 14, in 10 mM Na_2_HPO_4_ pH 7.4, 50 mM NaCl, 1 mM (TCEP), 10% D_2_O, 0.02% NaN_3_. Some non-specific signal loss is observed during the titration of RRM12 but not of the other di-domains, most likely because of a small amount of aggregation. For ^15^N-KH34, an additional equivalent titration was performed in the same buffer but higher salt concentration (150 mM NaCl). All NMR spectra were processed using NMRpipe [26] and analysed with CCPN [27] and TopSpin 4.0.6 (Bruker) software. Chemical shifts perturbations (CSP) were calculated using the formula:

$$CSP=\sqrt{{\left(\delta_{{\;}_{\;}^{1}H}\right)}^{2}+0.15{\left(\delta_{{\;}_{\;}^{15}N}\right)}^{2}}$$

where δ_1H_ and δ_15N_ are the chemical shift differences of the ^1^H and ^15^N dimensions, respectively. The published ^15^N-HSQC resonance assignments [28] were obtained from the Biological Magnetic Resonance Data Bank database and transferred to our ^15^N-HSQC spectra.

### Sequence and protein alignments

Primary sequence alignments of KH12 and KH34 were carried out with T-COFFEE multiple sequence alignment server [29] and alignment figures were generated using Jalview [30] using the CLUSTAL X conservation representation. Protein structure alignments were computed in DALI [31], using the previously reported structures of KH12 and KH34 (PDB access used: 6QEY [21], 3KRM [32], 2N8M, 2N8L [33]). All the structure images were obtained with PyMOL Molecular Graphics System 2.0 (Schrödinger, LLC).

### Statistics and reproducibility

All MST experiments were repeated at least three times, with every point performed in triplicate. K_D_ values are reported with standard deviation. FP experiments for the HTS (greater than 27,000 compounds) were performed once (with an overall Robust Z’ factor of 0.53); 504 molecules showed a standard deviation greater than 3. Of these, 48 compounds passed a further quality control screen (see text) and then were tested in a dose-response assay performed in triplicate in parallel to a counterscreen of an unrelated protein/RNA pair using the same FP reaction. Seven molecules demonstrated specific, dose-dependent inhibition of Igf2bp1-Kras 6 RNA binding. One molecule, 7773, was resynthesized twice and inhibited Igf2bp1 binding of Kras 6 RNA with an IC_50_ of ~30 μM. EMSA experiments were repeated 3 times, and representative gels are shown. Three biological repeats were used for each point in the wound healing and proliferation assays, and each experiment was performed at least twice. For each RT-PCR experiment, 3 biological repeats were performed, with 3 technical repeats for each sample. Western blots of phosphoERK and Kras proteins were performed twice, with 2 biological repeats for each point. Growth in soft agar was performed twice, with 3 biological repeats for each experimental group.

### Data availability

In accordance with the UK Medical Research Council policy, the authors will make available data, software and materials related to this study. Plasmids for the expression of IMP1 RRM12, KH12, and KH34 are available from the authors upon request.

## Results

### Assay for Kras RNA-Igf2bp1 binding

**Figure 1.**
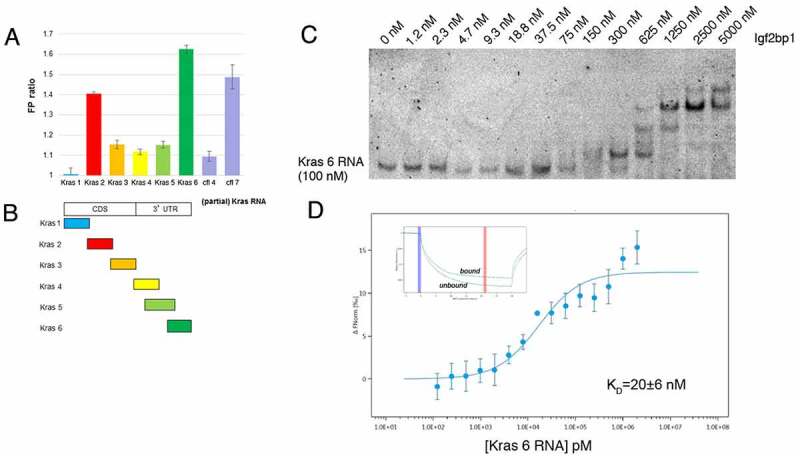
Identifying a fragment of Kras mRNA to probe for Igf2bp1 binding. (a) Different fragments spanning the Kras mRNA coding region, and part of its 3ʹUTR, were 5ʹ end labelled with fluorescein and used as substrates for Igf2bp1 binding in an FP assay. Two fragments from cofilin mRNA, cfl7 and cfl4, that were previously shown to bind or not bind, respectively, Igf2bp1 [36], were included as controls. (Y axis, FP ratio; no shift-i.e. no binding – FP ratio = 1.) (b) Schematic map of the different fragments of Kras used in the FP assay in (a). (c) Increasing concentrations of Igf2bp1 protein (indicated at the top of the lanes), incubated with 100 nM fluorescently labelled Kras 6 RNA, retarded migration of the RNA in a non-denaturing polyacrylamide gel (EMSA). (d) The kinetics of Kras 6 RNA binding to Igf2bp1 were monitored by MST; insert, a representative MST thermophoresis curve.

Kras^G12V^ is known to be a driver mutation for lung adenocarcinomas [34], and we have shown that Igf2bp1 not only binds Kras RNA in mouse lung carcinoma (LKR-M) cells but also synergizes with mutant Kras in promoting human lung adenocarcinomas [11]. Previous work indicated that Igf2bp1 binds the 3ʹUTR of Kras RNA [10,18]. We therefore developed an HTS assay to look for small molecules that would inhibit Igf2bp1 binding to Kras RNA, in order to identify molecules that could have therapeutic benefit in preventing Igf2bp1-mediated adenocarcinomas. We synthesized and fluorescently labelled RNA fragments that spanned the coding region and beginning of the 3ʹUTR of Kras RNA and tested these probes for their ability to bind Igf2bp1 using an FP assay. This homogeneous and rapid technique measures the degree to which a fluorescent molecule is sequestered in solution upon binding to another molecule [35]. As seen in Fig. 1(A,B), one fragment in the 3ʹUTR, Kras 6, bound Igf2bp1 with high affinity, even when compared with a control RNA fragment from cofilin mRNA (cf7), previously shown to be a functional Igf2bp1 target [36]. Igf2bp1 binding to the Kras 6 fragment was also tested using an electrophoretic mobility shift assay (EMSA, Fig. 1C). As protein concentration increased, an initial high-affinity binding event was followed by the appearance of several migration intermediates, suggesting additional recruitment of Igf2bp1 molecules to the RNA-protein complex. This multimerization of Igf2bp1 on RNA had been previously reported for other target RNAs [18,37]. As expected, the inclusion of increasing amounts of unlabelled Kras 6 RNA in the binding reactions inhibited the formation of the protein-RNA complexes seen in the EMSA assay (Supplemental Fig. 1A), and a similar inhibition of binding was observed in an equivalent FP assay (Supplemental Fig. 1B). In contrast, competition with unlabelled Kras 2 RNA was less efficient at inhibiting the Igf2bp1-Kras 6 RNA interaction (Supplemental Fig. 1A). These results confirm that Kras 6 contains a high-affinity binding site for Igf2bp1, as observed in our FP experiments, although Kras 2 may represent a secondary binding site, albeit with lower affinity.

Microscale thermophoresis (MST) is a quantitative method for assessing interactions between molecules [38]. Differential migration of fluorescent molecules in an induced thermal gradient can be used to determine the strength of the interaction between Igf2bp1 and an RNA target. We fluorescently labelled the poly-histidine tag of recombinant Igf2bp1-His tag protein and analysed its binding to unlabelled Kras 6 mRNA using MST. In addition to confirming the interaction between Igf2bp1 and Kras 6 mRNA, we found that the dissociation constant (K_D_) between Igf2bp1 and Kras 6 mRNA is ~20 nM (Fig. 1D). These results are consistent with the binding strength observed in our EMSA assay (Fig. 1C), even though, in the case of the MST experiment, it is the protein, and not the RNA, that is fluorescently labelled.

Igf2bps contain 6 putative RNA binding domains, arranged in di-domains from the N- to C-terminus: RRM (*RNA Recognition Motif*) 1 and 2, KH (*hnRNP-K homology domain*) 1 and 2, and KH3 and 4. Two of these di-domains, KH12 and KH34, were shown to bind to a range of RNA cognate sequences *in vitro* and are required for the functional interaction with different targets in cells [21,28,32,33,39]. It is noteworthy that the functional interaction of Igf2bp1 with some targets, such as β-actin, requires only the KH34 di-domain, while for the interaction with other targets, both KH12 and KH34 are required. To the best of our knowledge, there is no report of the RRM12 di-domains being essential for the interaction with physiological RNA targets. To characterize which of the RNA binding di-domains of the protein binds Kras 6 RNA, we fluorescently labelled peptides containing the different di-domains and tested these in the MST assay. As shown in Supplemental Fig. 3, Kras 6 RNA bound only to KH34, with a K_D_ of ~200 nM. This affinity is only ten-fold weaker than that of the entire protein, and, as no binding to the other isolated di-domains is observed, these data suggest that the key interactions between Igf2bp1 and Kras 6 RNA are mediated by KH34. The higher affinity of the full-length protein indicates that additional, weaker interactions with the other domains are also possible. As for many other Igf2bp1 targets in highly proliferating cells, the exact binding site of KH34 is difficult to define, with multiple putative imperfect sites for the individual KH domains present in the Kras6 sequence.

### HTS for small molecule inhibitors of Igf2bp1

**Figure 2.**
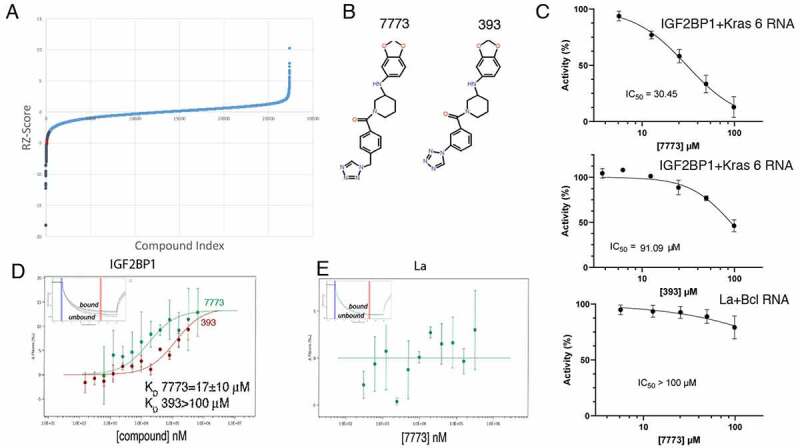
High throughput screen for Igf2bp1 inhibitors. (a) A snake plot of the FP screen for inhibitors of Igf2bp1 binding to fluorescent Kras 6 RNA. Over 27,000 compounds were screened (X-axis), and the robust Z score for each assay was plotted along the Y-axis. The shaded points on the curve represent compounds that showed a fluorescence polarization shift of greater than 3 standard deviations; the red point indicates the position of compound 7773. (b) The structure of compounds 7773 and 393. (c) 7773 or 393 was added at increasing concentrations (X-axis), and the percent activity of protein-RNA binding was plotted on the Y-axis. For Igf2bp1 binding to Kras 6 RNA, the IC_50_ for 7773 was 30.45 μM (N = 4), and for 393, the IC_50_ was 91.1 μM (N = 1). As a control, the ability of 7773 to inhibit binding of a different RNA binding protein, La, to Bcl2 RNA was assayed, and its IC_50_ was greater than 100 μM (N = 3). (d) MST was used to determine the K_D_ of Igf2bp1 binding to either 7773 (green) or 393 (red). (e) No binding of 7773 to La was observed in an MST assay. Representative MST thermophoresis curves are inserted in (D) and (E).

Having identified and characterized a fragment of Kras RNA that binds Igf2bp1, we miniaturized the FP assay to a 1536-well format in order to screen approximately 27,000 compounds (Fig. 2A). The screen was robust, with an overall Robust Z’ factor of 0.53. Five hundred and four compounds caused an FP shift of 3 standard deviations or greater. After removing molecules with low or high total fluorescence and correcting for technical plate and assay trending, promiscuity in other assays, and low chemical tractability, 48 compounds were selected for further confirmation by repeat assays and counterscreening of an unrelated protein/RNA pair using a similar FP reaction. Seven molecules demonstrated specific, dose-dependent inhibition of Igf2bp1-Kras 6 RNA binding. One molecule, 7773 (Fig. 2B), was resynthesized and inhibited Igf2bp1 binding of Kras 6 RNA with an IC_50_ of ~30 μM but had almost no effect on a control RBP (La) binding to its target (Bcl2 RNA) (Fig. 2C) or on a histone methyltransferease protein, G9a, catalysing methylation of a peptide (Supplemental Fig. 3). A very similar molecule, 393 (Fig. 2B), showed mild inhibition of Igf2bp1-Kras 6 RNA binding, with an IC_50_ of ~90 μM (Fig. 2C). These molecules were used for further studies.

(Fig. 3)

**Figure 3.**
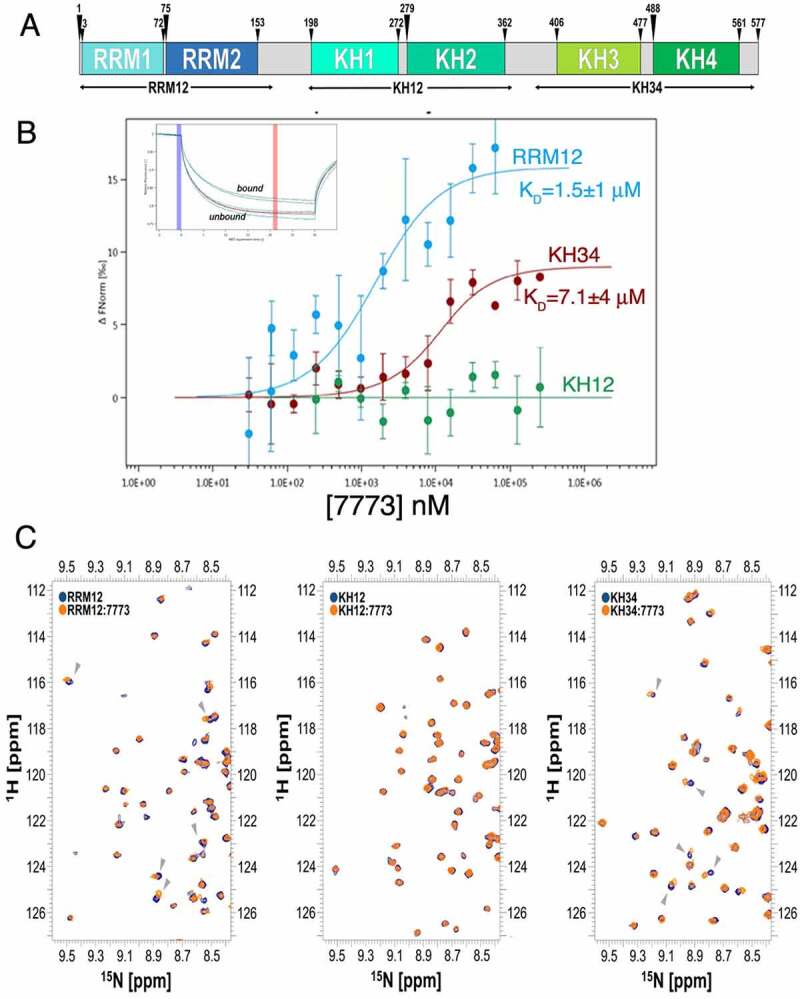
7773 binds to RNA binding domains of Igf2bp1. (a) Domain structure organization of Igf2bp1. Six RNA binding domains are organized in three di-domains: RRM12, KH12 and KH34. The arrowheads indicate the boundaries of the individual RNA binding domains; the di-domain constructs used in this study are indicated by arrows below the map. (b) MST binding curves of 7773 to the di-domains RRM12 (blue), KH12 (green), and KH34 (red). Representative MST thermophoresis curves are inserted. (c) Overlay of ^15^N-HSQC spectra recorded on the free RRM12, KH12 and KH34 di-domains (navy) and at a 1:14 protein-to-7773 molar ratio (Orange). The dispersed region between ^1^H 8.3–9.6 and ^15^N 111.6–127.2 ppm is displayed, as representative of the full spectra of the titration reported in Supplemental Fig. 3. Grey arrowheads indicate peaks that shifted significantly upon addition of 7773.

### *In vitro* validation of Igf2bp1 RNA binding inhibition

We made use of the MST assay to determine the kinetics of the interaction between the inhibitors, 7773 and 393, and Igf2bp1 protein. As seen in Fig. 2D, increasing concentrations of 7773 cause a significant shift in the curve, yielding a calculated K_D_ of 17 μM. When the less effective 393 compound was used, an approximately seven times lower affinity was observed (K_D_ of ~120 μM). The K_D_ values calculated from the MST data are consistent with the IC_50_’s obtained from the dose response FP curves and confirm a direct interaction between the inhibitors and Igf2bp1. Compound 7773 showed no binding in the MST assay to the control RNA binding protein, La (Fig. 2E).

To identify which of the Igf2bp1 structural units may interact with 7773, we tested the ability of the compound to bind the various di-domains using MST. 7773 interacts with both RRM12 and KH34 with K_D_ values of 1.5 and of 7.2 µM, respectively (Fig. 3B). These affinities are similar or higher than the one measured for the full-length protein (17 µM), consistent with the compound’s binding independently to the individual di-domains. This may result in an underestimate of the *K_D_*. It is also worth mentioning that, although the intact protein binds the compound with marginally lower affinity than the individual di-domains, it is possible that the interaction surface is more exposed in the individual domains, increasing their affinity slightly. In addition, it is possible that the interaction surface is more exposed in the individual domains. Interestingly, no binding was observed to KH12.

The interactions above were validated using ^15^N-HSQC NMR experiments, where the amide proton signals of the individual di-domains were monitored by titration with increasing amounts of 7773. Changes in position and intensity of the peaks are diagnostic of perturbations of the microenvironment of the amide groups and can be used to evaluate binding. While we observed peak shifts of several amide protons in the RRM12 and KH34 titrations, we did not detect similar changes in the KH12 titration (Fig. 3C; Supplemental Fig. S4). In corroboration with the results from the MST experiments, these data indicate that the 7773 compound interacts *in vitro* with the RRM12 and the KH34, but not the KH12, di-domains

**Figure 4.**
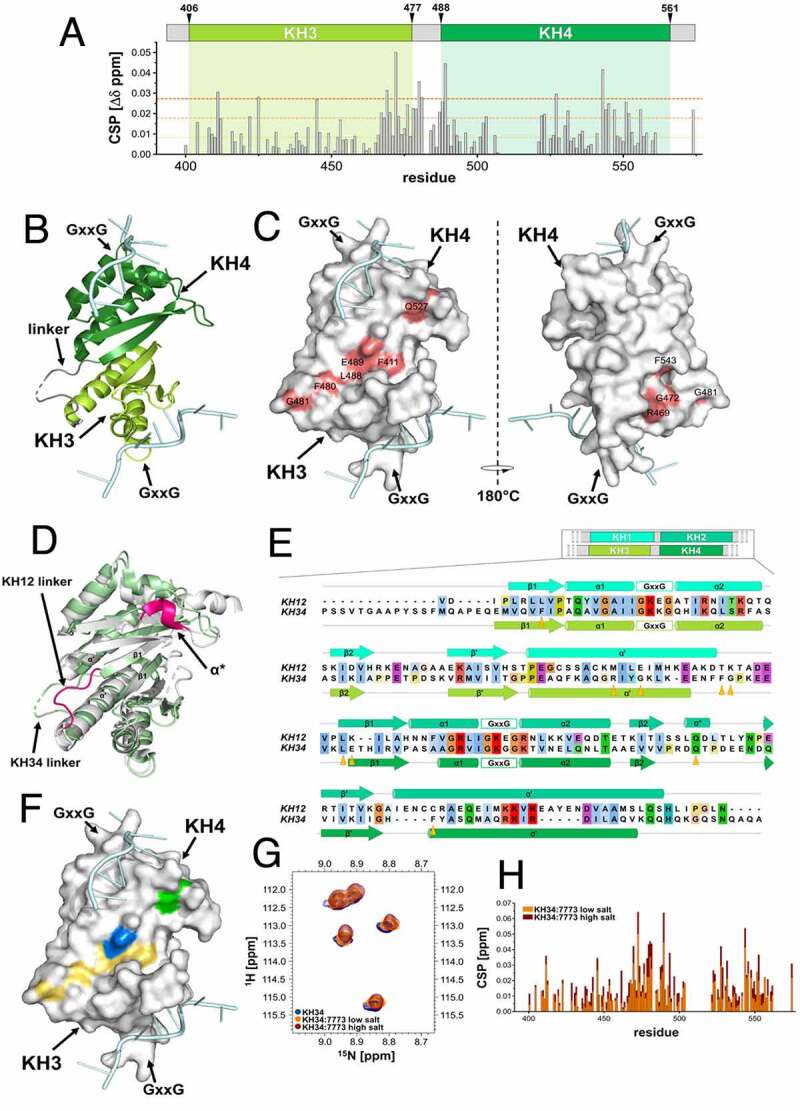
7773 binding surface. (a) KH34 per-residue chemical shift perturbations (CSP) recorded upon 1:147,773 binding. The green boxes indicate the KH3 and KH4 domains boundaries. The dotted lines indicate, respectively, one (yellow), two (Orange) and three (red) standard deviations of the average CSP observed. (b) Cartoon representation of KH34 di-domain. RNA is coloured in pale cyan. (c) Binding surface of 7773 on KH34. The affected residues, in the space filling model, are depicted in salmon, with the left panel oriented as in (B), and the right panel with a 180° rotation. (d) DALI Structural alignment of Igf2bp1 KH12 (grey) and KH34 (pale green) di-domains. The structural elements that are not conserved between the two di-domains are highlighted in magenta. (e) Primary sequence CLUSTAL X alignment of KH12 and KH34. The secondary structure elements are reported above and below KH12 and KH34 sequences, respectively. The residues of the binding surface are indicated by Orange arrowheads. (f) KH34 hydrophobicity. The hydrophobic, polar neutral and charged residues are depicted, respectively, in yellow, green and blue, while the RNA in pale cyan. The panel is oriented as in (B). (g) Overlay of the ^15^N-HSQC spectra of KH34 in the apo and complex with 7773 in low and high salt conditions (navy, Orange, and maroon, respectively). The panel reports a zoom in the representative region between ^1^H 8.66–9.10 and ^15^N 111.50–116.00 ppm, as representative of the full spectra of the titration reported in Supplemental Fig. 4. (h) KH34 per-residue chemical shift perturbations (CSP) recorded upon 1:147,773 binding in low (Orange) and high (maroon) salt.

Knowing that Kras 6 RNA binds only to the KH34 domain, we wanted to identify the 7773 binding surface on KH34. We thus transferred the existing assignment of Igf2bp1 KH34 backbone amide [28] onto our ^15^N-HSQC spectra (Supplemental Fig. 5) and identified the amide-protons’ resonances that shift upon addition of 7773 (Fig. 3C; Supplemental Fig. 5). To map the binding site of 7773 onto KH34, we focused on those residues that exhibited a chemical shift perturbation (CSP) above three standard deviations of the average CSP observed (Fig. 4A). The mapping of these onto the structure of KH34 (Fig. 4B) allowed us to visualize the residues whose amide groups’ microenvironment is perturbed the most by the inhibitor. We could therefore build a main 7773 binding site onto KH34 (Fig. 4C), an elongated surface at the interface between KH3 and KH4, involving mostly residues of the α’ and β1 of both domains, as well as amino acids in the inter-domain linker. A few more residues were found to significantly shift outside, but close to, the main binding site. The perturbation of these residues might indicate a direct interaction with a chemical moiety of 7773, but could also result from a small conformational rearrangement due to the binding of the molecule onto the KH34 di-domain. Significantly, our data indicate that 7773 binds outside the canonical RNA interacting grooves, the GxxG motifs [40].

Interestingly, despite the similar inter-domain arrangement and overall structural features of the di-domains [21,33], 7773 binds to KH34 but not KH12. In order to understand this difference, we compared the structural features of the two di-domains. Although in both KH12 and KH34, the inter-domain interface is mediated by the interaction of residues of the six-stranded beta-sheets β1 and the alpha helices α’, the conformation of the inter-domain linker, which is a key part of the 7773 interaction surface, is very different. In KH12, the shorter linker is stretched in a linear conformation that spans the distance between KH1 α’ and KH2 β1, while in KH34 it traces a wide turn that allows formation of a small hydrophobic core. Presumably related to the differences in the arrangement of the linker, the angle between the individual KH domains is different in KH12 and KH34, with the KH3 and KH4 domains creating a more open, planar, inter-domain surface [21]. Moreover, KH2 presents a non-conventional one-turn helix α* between β2 and β1 which, although outside of the main binding area, may contribute to occlude the 7773 binding surface (Fig. 4D). In addition to the above structural differences, most of the residues in the interaction surface are different in KH12 and KH34. The mapping of the 7773-dependent chemical shift changes on the sequence alignment of the KH12 and KH34 indicated that seven of the nine significantly perturbed residues are different in the two di-domains (Fig. 4E). The analysis of the structure and amino acid composition of the 7773-KH34 interaction surface together explain the difference in binding between KH12 and KH34.

We also evaluated the overall properties of the 7773-KH34 interaction surface. An analysis of residue type (polar, charged and hydrophobic) revealed that the inhibitor-binding region is predominantly hydrophobic (Fig. 4F). To validate this observation and assess the contribution of the hydrophobic contacts to binding, we probed the salt-dependency of the interaction in ^15^N-HSQC KH34 experiments. We titrated 7773 into a KH34 sample in a buffer with a higher but physiological salt concentration and compared the results with the lower salt titration discussed above (Fig. 4G and Supplemental Fig. 6). Comparison of the chemical shift changes caused by 7773 binding at 50 mM and 150 mM salt showed that even a relatively modest three-fold increase in salt concentration shifted the protein resonances towards the bound position (25% on average) (Fig. 4H), which is best visualized by plotting the shift of the representative G472 residue during the titration (Supplemental Fig. 6). This increase in binding affinity confirms the importance of the hydrophobic residues and provides a general insight into the forces driving the interaction.

**Figure 5.**
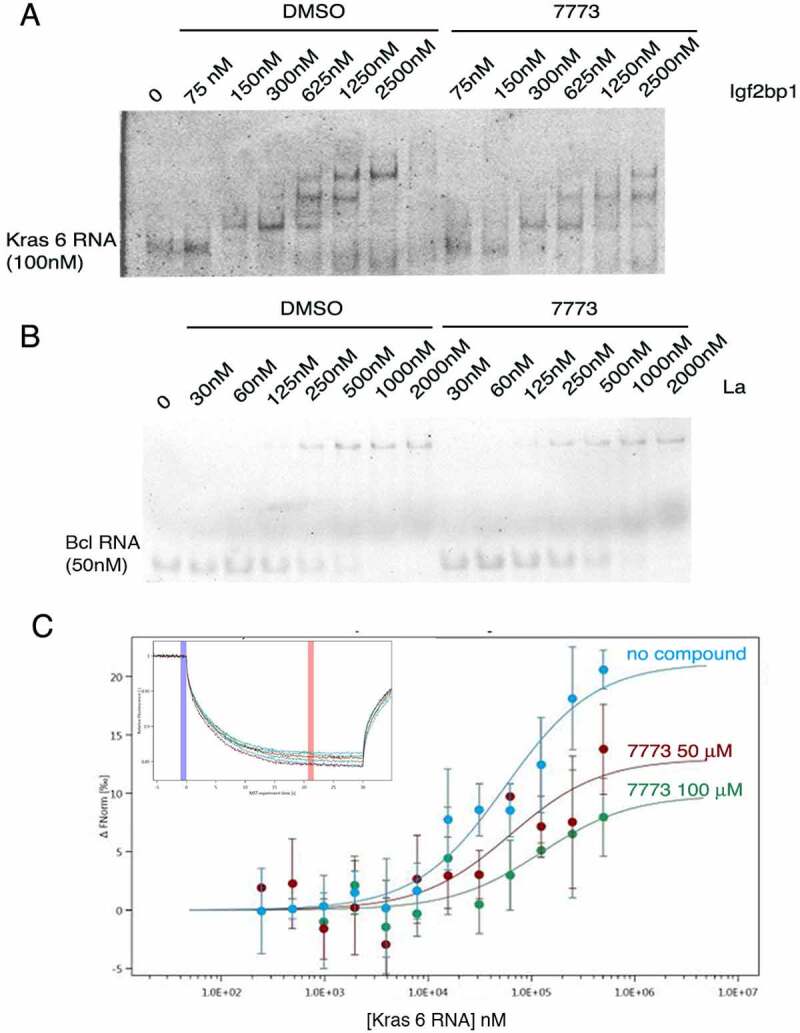
Inhibition of Igf2bp1 RNA binding *in vitro*. (a) EMSA with fluorescent Kras 6 RNA comparing the effect of incubation of increasing concentrations of Igf2bp1 with either DMSO or 200 μM 7773 on retardation of RNA migration. (b) EMSA with fluorescent Bcl2 RNA comparing the effect of incubation of increasing concentrations of La protein with either DMSO or 200 μM 7773 on retardation of RNA migration. (c) MST analysis of the effect of 7773 on the binding of Igf2bp1 to Kras 6 RNA. In the absence of compound, the K_D_ = 56 nM; in the presence of 50 μM 7773, the K_D_ = 63 nM, and in the presence of 100 μM 7773, the K_D_ = 120 nM. Representative MST thermophoresis curves are inserted.

To validate that 7773 disrupts Igf2bp1 binding to its RNA target, we made use of both EMSA and MST assays. Igf2bp1 protein was pre-incubated with either 7773 (dissolved in DMSO) or DMSO and then tested for its ability to bind Kras 6 RNA, as assayed by gel shift. As Igf2bp1 concentrations are increased, slower-migrating RNA-protein complexes appear on the gel in a protein concentration-dependent manner, even in the presence of DMSO (compare Fig. 5A to Fig. 1C). In the presence of the inhibitor 7773, however, a clear reduction in slower-migrating bands is observed at all of the protein concentrations tested. The 393 inhibitor, which showed a much lower affinity for binding to Igf2bp1 in the MST assay, exhibited a lower, but observable, inhibition of RNA binding in the EMSA assay (Supplemental Fig. 7). Consistent with the results observed with FP, neither 7773 nor DMSO affected the ability of La protein to retard the mobility of its target RNA, Bcl2 (Fig. 5B). It is worth noting that La binds RNA mainly via a La domain and an RRM domain. La protein therefore acts both as a general control and an ‘RRM fold’ control in the assays above.

To further verify the activity of 7773, we tested its ability to inhibit Igf2bp1 binding to Kras 6 RNA in a dose-dependent manner using the MST assay. As seen in Fig. 5C, increased concentrations of 7773 led to smaller shifts in the thermophoresis curves, as expected from the reduction in the number of Igf2bp1 molecules, not complexed with 7773, that are available to bind Kras 6 RNA. These results validate those obtained from the FP, NMR, and EMSA assays and suggest that 7773 is a direct, effective, and selective inhibitor of Igf2bp1 RNA binding *in vitro*.

### Inhibition of the other Igf2bp paralogues

**Figure 6.**
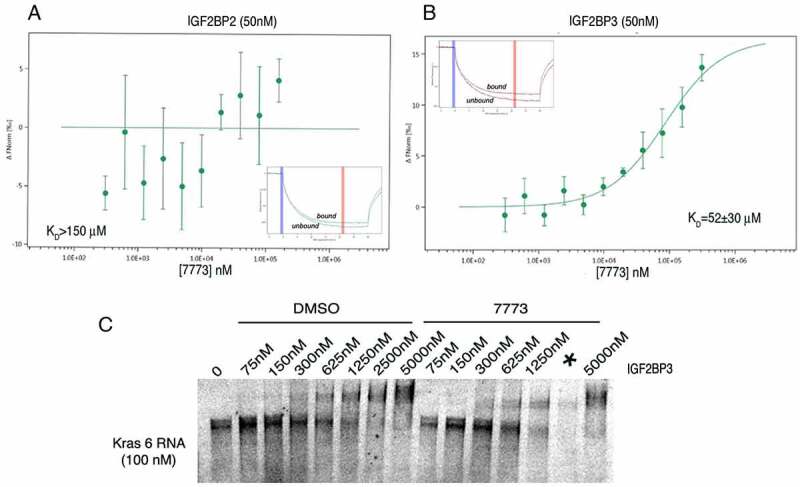
Binding of 7773 to other Igf2bp paralogues. MST analysis of 7773 incubation with (a) Igf2bp2 and (b) Igf2bp3. Representative MST thermophoresis curves are inserted. (c) EMSA with fluorescent Kras 6 RNA comparing the effect of incubation of increasing concentrations of Igf2bp3 with either DMSO or 200 μM 7773 on retardation of RNA migration. *, indicates that some of the sample leaked out of the lane.

Given the high degree of similarity among the Igf2bp paralogues, we explored the specificity of 7773 for Igf2bp paralogs using the MST assay. 7773 did not significantly bind Igf2bp2, even at concentrations that almost saturate the binding to Igf2bp1 (Fig. 6A). 7773 did bind Igf2bp3 (Fig. 6B), although the K_D_ was significantly higher (52 μM) than that observed with Igf2bp1 (17 μM). The gel shift assay also confirmed that 7773 weakly inhibited Igf2bp3 binding of Kras 6 RNA compared to the DMSO control (Fig. 6C). These results indicate that 7773 inhibits *in vitro* Kras RNA binding of both Igf2bp1 and Igf2bp3, but not Igf2bp2.

### 7773 targets Igf2bp1 activity in cells

**Figure 7.**
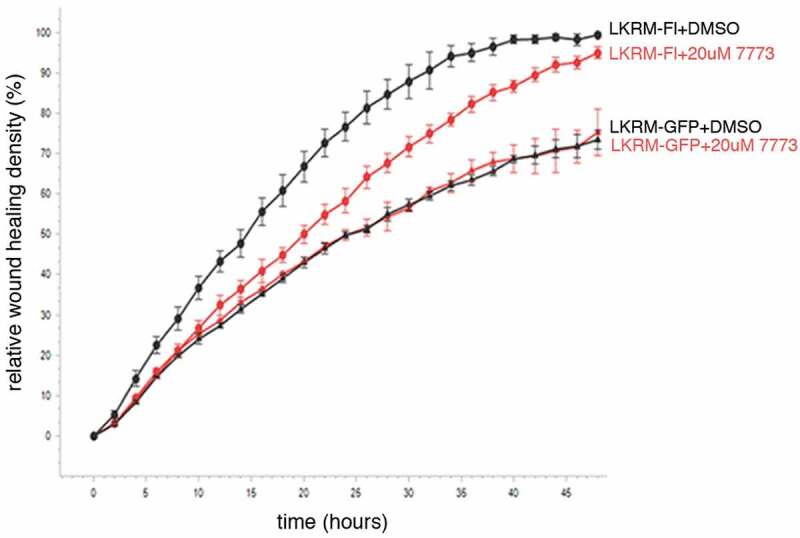
7773 targets human Igf2bp1 in transfected mouse LKR-M cells. LKR-M cells were transfected with either full length human Igf2bp1-GFP (*Fl*, circles(or GFP alone (*GFP*, triangles) and then assayed by Incucyte Live Cell Analysis System for their ability to migrate in a wound healing assay in the presence of either 20 μM 7773 (red) or DMSO (black).

We made use of two different assays to test whether 7773 also targets Igf2bp1 in cultured cells. First, we followed how treating cells with the compound effects their migration in a wound healing assay. Previous work demonstrated the involvement of Igf2bp1 in helping mediate cell migration in lung adenocarcinoma cells, both in cultured cells and in mice [11]. Here, mouse LKR-M cells overexpressing either GFP or human Igf2bp1-GFP were cultured with either DMSO or 7773. As seen previously, exogenous human Igf2bp1-GFP enhanced wound healing when compared to the expression of GFP alone (Fig. 7). This enhancement was significantly inhibited by exposure of the Igf2bp1-GFP-expressing cells to 7773. The migration of LKR-M cells expressing GFP alone, however, was unaffected by incubation with 7773 (as compared to DMSO), suggesting that there were no off-target effects of 7773 incubation. Notably, the only Igf2bp paralogue endogenously expressed in LKR-M cells is Igf2bp2 [11], which is not bound by 7773 *in vitro* (see Fig. 6). These results indicate that human Igf2bp1 activity, which helps mediate cell migration when expressed in LKR-M cells, is directly and specifically targeted and inhibited in these cells by incubation with 7773.

**Figure 8.**
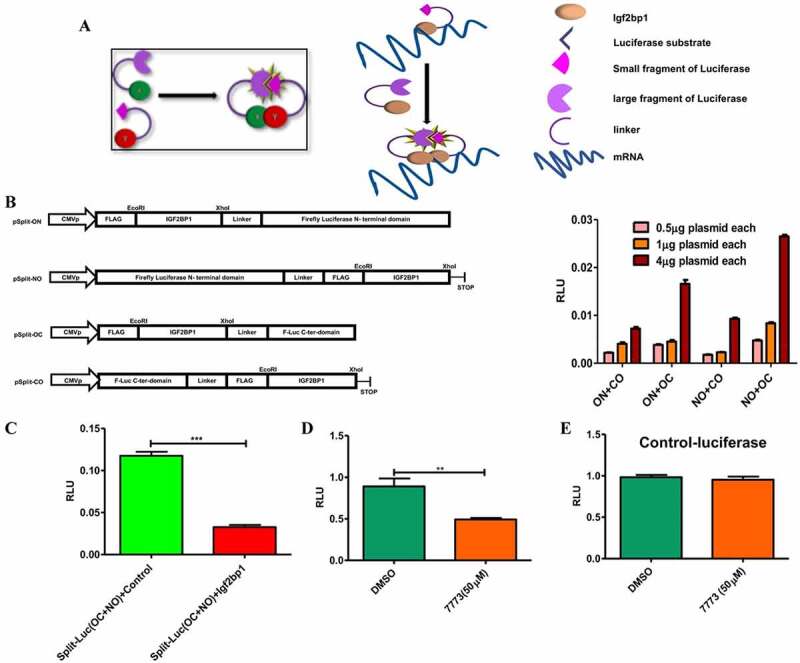
Use of a split-luciferase assay to observe 7773 targeting of Igf2bp1 in transfected RKO cells. (a) Schematic presentation of assay principal: luciferase activity is detected only when Igf2bp1 dimerizes on target RNA. (b) Four split luciferase reporter plasmids fused with Igf2bp1 in different orientations vis-à-vis the luciferase fragments (left) were assessed for efficiency of luciferase activity in RKO cells (right). (c) Ectopic expression of wild-type Igf2bp1 inhibits split-luciferase activity. Treatment with 50 μM 7773 inhibited (d) split-luciferase activity, but not (e) control luciferase in RKO cells.

As a second test for direct 7773 targeting of Igf2bp1 in cells, we developed an assay based on the requirement of Igf2bp1 to dimerize in order to stably bind RNA [37]. Vectors expressing fragments of a split luciferase reporter with a flexible linker [20] were fused to Igf2bp1 in different orientations, and the combination and concentration that gave optimal luminescence was identified by transfection into RKO cells (that express very low levels of Igf2bp1 [41];). When dimerization of the Igf2bp1-luciferase reporter fusions was inhibited by co-transfection of an Igf2bp1 overexpression plasmid, luminescence was significantly reduced compared to co-transfection with a control plasmid (with no insert;). This result was consistent with previous reports demonstrating the presence of Igf2bp1 dimers/multimers in RNP complexes and validates the utility of our reporter system for detecting these interactions. Incubation with the 7773 inhibitor significantly reduced luminescence in RKO cells transfected with the luciferase reporter fusions, indicating that the compound was targeting Igf2bp1 in these cells. Importantly, 7773 did not affect the activity of wild-type luciferase, demonstrating its specificity for Igf2bp1. (Fig. 8)

### 7773 inhibits neoplastic activity

**Figure 9.**
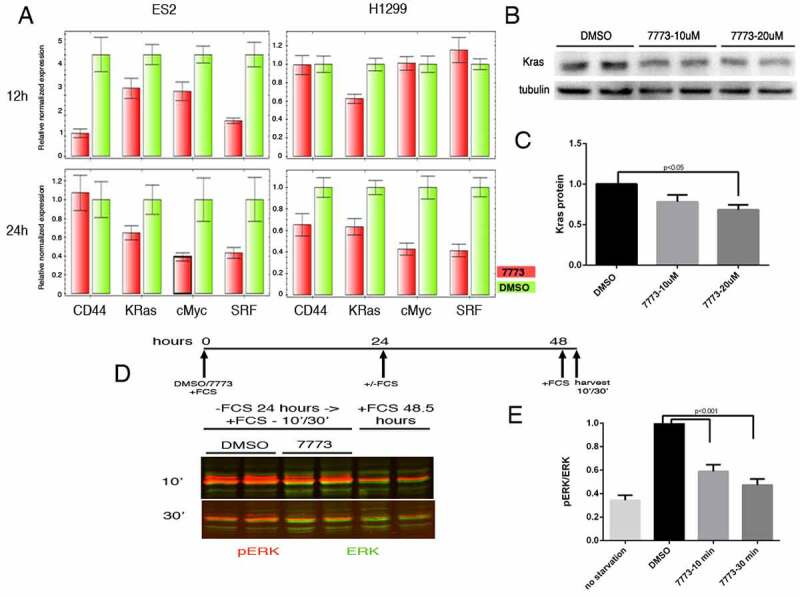
7773 downregulates Kras and other Igf2bp1 target RNAs, Kras protein, and downstream signalling. (a) Real-time PCR analysis of steady state levels of CD44, Kras, cMyc, and SRF RNA in ES2 or H1299 cells treated with either 20 μM 7773 (red) or DMSO (green) for 12 or 24 hours. (b) Western blot analysis of Kras expression in H1299 cells incubated in 20 μM 7773, 10 μM 7773, or DMSO for 48 hours. Duplicates were performed and analysed for each concentration. Tubulin was used as loading control. (c) Quantification of the blot in (B), normalized to tubulin and to the relative level of Kras protein in the DMSO-treated samples. (d) H1299 cells were cultured with either 20 μM 7773 or DMSO for 24 hours, after which FCS was removed from the medium and the cells were cultured for another 24 hours. At that point, FCS was re-added for either 10ʹ or 30ʹ and then protein extracts were prepared and analysed by Western blot for expression of ERK (green) and phosphoERK (pERK, red). Cells grown continuously in FCS for 48.5 hours were used as controls. Experimental duplicates were run on the gel. (e) Quantification of the western shown in (D), with the ratio of pERK to ERK plotted on the Y-axis.

Igf2bp1 has been shown to stabilize a number of its RNA targets through binding to either coding or non-coding sequences in the mRNA [16]. To see whether 7773 inhibition of Igf2bp1 affects target RNA levels, we analysed steady state levels of several previously identified, cancer-associated RNA targets in ES2 and H1299 cancer cell lines, which express high levels of Igf2bp1 [9,36], using quantitative PCR (Fig. 9A). Incubation of cells with 7773 for either 12 or 24 hours caused a clear reduction in steady state levels for all the RNAs assayed, although the degree and timing of the reduction was both cell-type and RNA dependent. In the ovarian carcinoma line, ES2, all of the assayed RNAs were reduced after 12 hours, with 3 of the 4 RNAs showing similar or even enhanced reduction after 24 hours. One of the RNAs, CD44, was strongly reduced after 12 hours, but appeared to recover after 24 hours. In the lung adenocarcinoma line, H1299, Kras mRNA was already reduced after 12 hours, with all of the RNAs significantly reduced after 24 hours. Control RNAs, which are not targets of Igf2bp1, were not significantly affected by incubation with 7773 after 24 hours in either ES2 or H1299 cells (Supplemental Fig. 8).

We tested whether the reduction in Kras RNA would lead to a reduction in Kras protein in the H1299 cells. Cells were incubated with DMSO or 7773 for 48 hours and then assayed for expression of Kras protein on a Western blot. Indeed, a dose-dependent reduction of Kras protein is observed (Fig. 9B,C). One readout of reduced Kras protein is downstream ERK signalling, which is associated with enhanced wound healing and cell migration [42]. The ratio of phosphorylated ERK (pERK) to total ERK in H1299 cells that were starved and then induced by addition of serum is also reduced by preincubation of the cells with 7773 (Fig. 9D,E). A similar reduction of pERK levels is observed in LKR-M cells expressing human Igf2bp1 (Supplemental Fig. 9). Taken together, these studies indicate that 7773 targets Igf2bp1 RNA binding in cells, leading to a reduction of Kras RNA, Kras protein, and pERK signalling.

**Figure 10.**
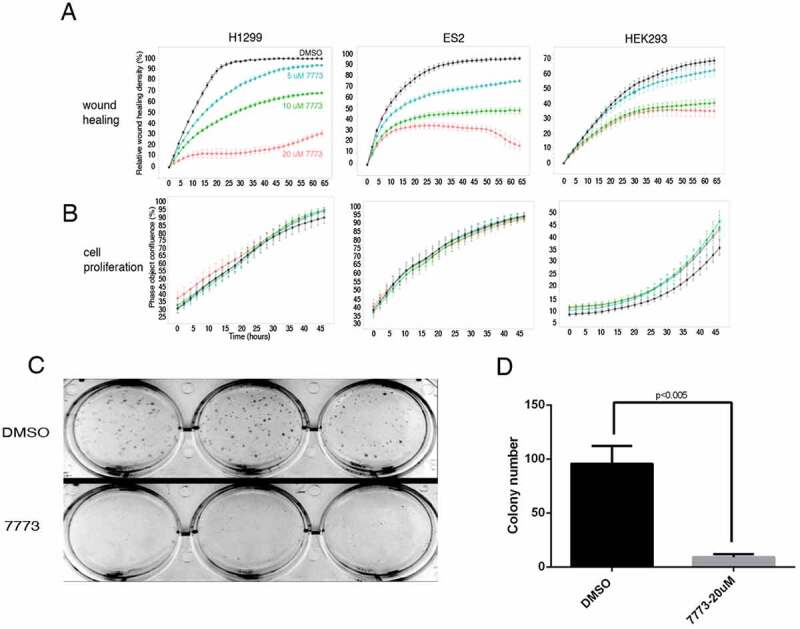
7773 inhibits wound healing and growth in soft agar without affecting cell proliferation. H1299, ES2, and HEK293 cells were incubated with DMSO (black) or different concentrations of 7773 (5 μM, blue; 10 μM, green; 20 μM, red) and analysed for (a) wound healing or (b) cell proliferation using the Incucyte Live Cell Analysis System. (c) H1299 cells were seeded in triplicate in soft agar in the presence of either DMSO or 7773 (20 μM) and cultured for 2 weeks. (d) Quantification of the results in (C).

After observing the inhibition of Kras signalling by 7773 on a molecular level, we followed the effects of a 2–3 day exposure to 7773 on cell migration and proliferation in three different human cell lines (H1299, ES2, and HEK293), expressing high levels of Igf2bp1 [2,11] (Fig. 10A). There was a pronounced repression of wound healing with increasing concentration of 7773; a discernible inhibition was observed at even the lowest concentration, 5 μM, in all of the cell lines. Cell proliferation in all the lines, as assayed by Incucyte, was remarkably unaffected, however, by even the highest concentration of 7773 (20 μM), a concentration that strongly inhibited wound healing in each of the lines (Fig. 10B).

Anchorage-independent growth is a hallmark of lung carcinomas and other neoplastic cells [43]. H1299 cells grow well when cultured in soft agar and form colonies from single cells that are observable by the eye after 2 weeks. Incubation of these cells in 20 μM 7773, however, dramatically inhibited their ability to form colonies in soft agar (Fig. 10C,D), despite virtually no effect on cell proliferation or cytotoxicity (Supplemental Fig. S10). Taken together, these results demonstrate that the effect of 7773 on lung adenocarcinoma cells is not the result of generalized toxicity but rather specific inhibition of signalling, migration, and growth associated with neoplastic cells.

## Discussion

Therapies directed at inhibiting Igf2bp1 function constitute a potentially powerful approach for fighting cancer, given the correlation between elevated Igf2bp1 expression and poor clinical outcomes [11,44], the activation of Igf2bp1 in a wide variety of cancers [1], and the effectiveness of preventing metastasis when Igf2bp1 activity is reduced [3,44–46]. Furthermore, Igf2bp1 is a very attractive target for therapy inasmuch as specific inhibitors would be expected to have minimal side effects: i) Igf2bp1 is expressed at very low levels in normal adult tissues [2], and ii) adult mice with an inducible whole-mouse knockout of Igf2bp1 in adult animals (utilizing newly generated UBC-Cre^ERT2^; Igf2bp1^loxP/loxP^ and Rosa26-Cre^ERT2^; Igf2bp1^loxP/loxP^ mice) are healthy (data not shown). For these reasons, we undertook a screen to identify small-molecule inhibitors of Igf2bp1. Evidence presented here indicates that a hit identified in the screen is a very promising lead molecule. *In vitro*, 7773 binds Igf2bp1 and inhibits its ability to bind Kras 6 RNA. When incubated with cells, 7773 targets Igf2bp1 and causes a reduction in Kras mRNA and other RNA targets, reduces Kras protein and downstream signalling, and represses cell migration and growth in soft agar. We anticipate that further refinement of this compound will lead to a new class of drugs that can be used in a clinical setting for treating lung adenocarcinomas as well as other neoplasias. We have previously described a role for Igf2bp1 in lung adenocarcinoma progression as well as in cell migration and metastasis [11]. In addition, Igf2bp1 has also been associated with enhancing cancer cell resistance to chemotherapy [8,47–51]. We thus envision that an optimized small molecule based on 7773 could be useful in a clinical setting, either as monotherapy directed against cancer progression, cell migration and metastasis formation or perhaps as adjuvant therapy in conjunction with chemotherapeutic agents.

Structural analysis of the binding of 7773 to Igf2bp1 has begun to shed some light on how this molecule functions. NMR analysis indicates that 7773 interferes with the binding of Igf2bp1 to its Kras RNA target by binding to a planar, elongated surface in the KH34 di-domain interface. Interestingly, binding is mainly hydrophobic but is not mediated by the two canonical base-recognition grooves, which are also hydrophobic. As shown previously, KH34 binds bipartite RNA sequences, with each domain contributing to the binding and the RNA spacer between the target sequences spanning the two canonical RNA-binding grooves. It seems possible that 7773 prevents RNA binding by occupying the path the RNA must trace to connect the two grooves. Alternatively, 7773 binding may result in local conformational changes in the residues framing the hydrophobic grooves. The results of the gel shift and MST experiments (Fig. 5) demonstrate that incubation of Igf2bp1 with 7773 shifts complex formation at higher protein concentrations, but also leads to a decrease of signal, indicating a complex effect on binding, possibly related to the use of multiple domains in protein-RNA recognition. Future work, including a higher resolution structural analysis of the interaction, followed by a mutational analysis, will help elucidate the exact mechanism of 7773 inhibition of mRNA binding.

Kras is the most commonly mutated oncogene in cancer and is thought to drive more than 30% of all tumours [17]. It has been a notoriously recalcitrant protein for targeted therapy. Here, we were able to obtain a reduction in steady-state Kras mRNA levels by treating lung and ovarian carcinoma cells with 7773 for 24 hours, and this reduction led to reduced Kras protein levels after 48 hours. ERK is a downstream effector of Kras signalling, undergoing phosphorylation and regulating several crucial cell functions, including proliferation and migration [42]. Incubation of lung adenocarcinoma cells with 7773 prior to activation of Kras signalling impairs ERK phosphorylation. Accordingly, treated cells also show a reduction of cell migration that is dependent on the concentration of the compound. Proliferation was strikingly unaffected by any of the concentrations of 7773, indicating that it is not toxic, although the ability of H1299 cells to grow in soft agar was severely impaired by incubation with the compound. Thus, 7773 treatment, by interfering with Igf2bp1 binding to Kras, appears to be a highly selective and effective tool for inhibiting Kras and at least some of its oncogenic downstream effects.

These data are consistent with those reported previously using a mouse lung adenocarcinoma cell line, LKR-M, in which either a human full length (FL-)Igf2bp1 or a dominant negative (DN-)Igf2bp construct was overexpressed. When these cells were xenografted subcutaneously into syngeneic mice, FL-Igf2bp1 overexpression significantly enhanced, and DN-Igf2bp overexpression significantly repressed, the number and burden of LKR-M derived lesions in the lungs. Proliferation of these cells, either *in vitro* or *in vivo*, was not significantly affected by the expression of either of the constructs. Wound healing, however, was increased by FL-Igf2bp1, and decreased by DN-Igf2bp [11] due to its ability to inhibit RNA binding of all the Igf2bp paralogs [52]. 7773 appears to mimic the effects of the DN-Igf2bp construct, with one notable exception, namely that 7773 does not inhibit wound healing in native LKR-M cells (Fig. 7). Significantly, these cells do not express endogenous Igf2bp1 but rather Igf2bp2, which is not bound by 7773 (Fig. 6). These results thus argue that 7773 targets Igf2bp1 selectively *in vivo* as well as *in vitro*. Selective targeting was further validated by the ability of 7773 to inhibit the split-luciferase reporter constructs fused to Igf2bp1, but not to control luciferase. Consistent with these findings is the fact that 7773 satisfies Lipinski’s Rule of 5 for drug solubility and cell uptake, having a molecular weight under 500 daltons and a cLogP of 2.5 (<5) [53].

Several other Igf2bp1 target RNAs (SRF, cMyc, and CD44) are reduced in lung and ovarian carcinoma cells incubated with the compound (Fig. 9). These results are consistent with the fact that Igf2bp1 appears to promote pro-oncogenic phenotypes based on its ability to bind many RNAs associated with neoplastic cells. SRF mRNA is protected from miRNA-mediated degradation via Igf2bp1 binding, and SRF and Igf2bp1 synergize to promote gene expression in cancer [54]. CD44 is a transmembrane glycoprotein whose aberrant expression is associated with invasion and metastasis in various cancers [55]. In HeLa cells, downregulation of Igf2bp1 and 3 causes an almost three-fold reduction in CD44 mRNA half-life and a loss of invadopodia [56]. cMyc is a transcription factor that regulates growth and proliferation of cells and is associated with a majority of human tumours [57]. Igf2bp1 binds a sequence in the coding region of cMyc mRNA that stabilizes it [58]. The importance of the Igf2bp1-cMyc mRNA interaction in tumorigenesis, however, remains to be determined, given that cMyc RNA is not upregulated in tumours induced by Igf2bp1 overexpression in the mammary glands of mice [59].

A small molecule that inhibited Igf2bp1-cMyc RNA interaction *in vitro* (termed BTYNB) has been identified using fluorescence polarization [60]. Although its mechanism of action, binding site, and specificity (vis-à-vis Igf2bp2, 3, or other RNA binding proteins) have not been described, this molecule appears to inhibit cell proliferation and reduce the level of a number of RNAs, similar to siRNA knockdown of Igf2bp1. 7773, with no obvious structural similarity to BTYNB, appears to have a much more selective effect on Igf2bp1 function. It shows high specificity for the Igf2bp1 paralog, does not affect proliferation, but does inhibit wound healing, ERK signalling, and growth in soft agar. Despite the downregulation, following 7773 treatment, of several RNAs associated with an oncogenic phenotype (Fig. 9A), the fates of the large majority of RNAs targeted by Igf2bp1 remain to be determined. Characterizing the global Igf2bp1 RNA binding changes (both down and up-regulated) that are induced by treatment with 7773 should provide insights into how the compound achieves its anti-tumour effects.

By virtue of their ability to bind a wide range of pro-oncogenic mRNAs, Igf2bp proteins represent an attractive target for directed therapies. Indeed, these proteins have been described as part of an epigenetic switch that occurs during oncogenesis [61]. Igf2bp proteins are also part of a select group of m6A readers [62], and methylation of RNAs is upregulated in many tumours, where it is often associated with oncogenic behaviour [63]. It is interesting to note that the small circular RNA circNDUFB2 has recently been shown to be inversely correlated with growth and metastasis in NSCLC patients. When expressed at elevated levels, this RNA facilitates ubiquitination and degradation of Igf2bps, and the process is enhanced when the circular RNA is methylated [64]. These results demonstrate the potential benefits of downregulating Igf2bp proteins by small molecules, and drugs that selectively inhibit these proteins can provide new approaches for fighting tumour progression and metastasis.
